# Clinicopathological profile and surgical treatment of abdominal tuberculosis: a single centre experience in northwestern Tanzania

**DOI:** 10.1186/1471-2334-13-270

**Published:** 2013-06-08

**Authors:** Phillipo L Chalya, Mabula D Mchembe, Stephen E Mshana, Peter F Rambau, Hyasinta Jaka, Joseph B Mabula

**Affiliations:** 1 Department of Surgery, Catholic University of Health and Allied Sciences-Bugando, Mwanza, Tanzania; 2 Department of Surgery, Muhimbili University of Health and Allied Sciences, Dar Es Salaam, Tanzania; 3 Department of Microbiology & Immunology, Catholic University of Health and Allied Sciences-Bugando, Mwanza, Tanzania; 4 Department of Pathology, Catholic University of Health and Allied Sciences-Bugando, Mwanza, Tanzania; 5 Department of Internal Medicine, Catholic University of Health and Allied Sciences-Bugando, Mwanza, Tanzania

**Keywords:** Abdominal tuberculosis, Clinicopathological profile, Outcome, Surgical treatment, Tanzania

## Abstract

**Background:**

Abdominal tuberculosis continues to be a major public health problem worldwide and poses diagnostic and therapeutic challenges to general surgeons practicing in resource-limited countries. This study was conducted to describe the clinicopathological profile and outcome of surgical treatment of abdominal tuberculosis in our setting and compare with what is described in literature.

**Methods:**

A prospective descriptive study of patients who presented with abdominal tuberculosis was conducted at Bugando Medical Centre (BMC) in northwestern Tanzania from January 2006 to February 2012. Ethical approval to conduct the study was obtained from relevant authorities. Statistical data analysis was performed using SPSS version 17.0.

**Results:**

Out of 256 patients enrolled in the study, males outnumbered females. The median age was 28 years (range = 16–68 years). The majority of patients (77.3%) had primary abdominal tuberculosis. A total of 127 (49.6%) patients presented with intestinal obstruction, 106 (41.4%) with peritonitis, 17 (6.6%) with abdominal masses and 6 (2.3%) patients with multiple fistulae in ano. Forty-eight (18.8%) patients were HIV positive. A total of 212 (82.8%) patients underwent surgical treatment for abdominal tuberculosis. Bands /adhesions (58.5%) were the most common operative findings. Ileo-caecal region was the most common bowel involved in 122 (57.5%) patients. Release of adhesions and bands was the most frequent surgical procedure performed in 58.5% of cases. Complication and mortality rates were 29.7% and 18.8% respectively. The overall median length of hospital stay was 32 days and was significantly longer in patients with complications (*p* < 0.001). Advanced age (age ≥ 65 years), co-morbid illness, late presentation, HIV positivity and CD4+ count < 200 cells/μl were statistically significantly associated with mortality (p < 0.0001). The follow up of patients were generally poor as only 37.5% of patients were available for follow up at twelve months after discharge.

**Conclusion:**

Abdominal tuberculosis constitutes a major public health problem in our environment and presents a diagnostic challenge requiring a high index of clinical suspicion. Early diagnosis, early anti-tuberculous therapy and surgical treatment of the associated complications are essential for survival.

## Background

Tuberculosis (TB) is a common and major health problem, especially in developing countries where, ignorance, poverty, overcrowding, poor sanitation and malnutrition are prevalent [1]. It has been declared a global emergency by the World Health Organization (WHO) and is the most important communicable disease worldwide [2,3]. Approximately one third of the world population is infected with tuberculosis and about three millions die each year from this disease [1-3]. Despite increased health standards in developed countries, the incidence of tuberculosis which was previously reported to be low in these countries, is again on the rise due to the influx of immigrants from third world countries, increasing incidence of human immunodeficiency virus (HIV) infection, an ageing population, alcoholism, increased use of immunosuppressive drugs, and the emergence of multi-resistant strains of *Mycobacterium tuberculosis*[4,5].

Most cases of TB are caused by *M. Tuberculosis* and the reservoir of infection is humans with active TB. Most cases of TB are pulmonary and acquired by person to person transmission of air-borne droplets of organisms. Abdominal TB may be contracted by drinking dairy milk contaminated with *M. Bovis*[6].

Tuberculosis can affect any part of the body and abdomen is the next common site after lungs affected by the disease [7]. In the abdomen, tuberculosis may affect the gastro-intestinal tract, peritoneum, lymph nodes, and solid viscera. Approximately 1-3% of total TB cases are extra pulmonary [8,9], of these abdominal tuberculosis (ATB) accounts for 11%-16% [10]. In HIV positive patients the incidence of extra pulmonary TB is up to 50% [1,10].

The modes of infection of abdominal tuberculosis include hematogenous spread from a primary lung focus that reactivates later or miliary tuberculosis, spread via lymphatics from infected nodes, ingestion of bacilli either from the sputum or from infected sources such as milk products, or by direct spread from adjacent organs [11].

Whereas intestinal (enteric) tuberculosis exists in one of the three main forms i.e. ulcerative, hypertrophic or ulcerohypertrophic, and fibrous stricturing form [12,13], peritoneal involvement (TB peritonitis) exists in four main forms namely ascitic, loculated (encysted), plastic (fibrous) and purulent forms [12]. The lymph nodes in the small bowel mesentery and the retroperitoneum are commonly involved, and these may caseate and calcify [13]. Disseminated abdominal tuberculosis involving the gastrointestinal tract, peritoneum, lymph nodes and solid viscera has also been described [11-13].

The diagnosis of abdominal TB in initial stages is difficult as the clinical features are vague, diverse and there is no specific diagnostic test [7,12,13]. It remains a considerable diagnostic challenge, especially in the absence of pulmonary infection, as the disease can mimic various gastrointestinal disorders, particularly the inflammatory bowel disease, colonic malignancy, or other gastrointestinal infections [7,12].

Abdominal tuberculosis is characterized by different modes of presentation, viz, chronic, acute and acute-on-chronic, or it may be an incidental finding at laparotomy for other diseases [14]. The clinical presentation depends upon the site and type of involvement. It usually runs an indolent course and presents late with complications especially acute or sub-acute intestinal obstruction due to mass (tuberculoma) or stricture formation in small gut and ileocaecal region or gut perforation leading to peritonitis [15].

The management of abdominal TB poses diagnostic and therapeutic challenges to general surgeons practicing in resource-limited countries such as Tanzania [1,12]. Late presentation of the disease coupled with ignorance, poverty, overcrowding, poor education, malnutrition and lack of modern diagnostic and therapeutic facilities are among the hallmarks of the disease in these countries. Despite advances in medical imaging, the early diagnosis of abdominal tuberculosis is still a problem and patients usually present when complications had occurred.

The treatment of abdominal tuberculosis is mainly conservative (non-operatively) with anti-tuberculous therapy and surgical treatment is reserved for complications such as intestinal obstruction and bowel perforation with peritonitis [12,16].

Despite the fact that abdominal tuberculosis and its complications is prevalent in our environment, little work on this subject has been done in Tanzania and the study area in particular. This study was conducted to describe the clinicopathological profile and outcome of surgical treatment of abdominal tuberculosis in our setting and compare with what is described in literature.

## Methods

### Study area and design

A prospective descriptive study of patients who presented to Bugando Medical Centre (BMC) with a clinical diagnosis of abdominal tuberculosis was conducted from January 2006 to February 2012. BMC is located in Mwanza city along the shore of Lake Victoria in the northwestern part of Tanzania. It is a 1000-bed, tertiary care and teaching hospital for the Catholic University of Health and Allied Sciences-Bugando (CUHAS-Bugando) and other paramedics. BMC is one of the four largest referral hospitals in the country and serves as a referral centre for tertiary specialist care for a catchment population of approximately 13 million people from neighboring regions.

### Study population and selection criteria

During the period of study, all patients who presented to BMC with clinical diagnosis of abdominal tuberculosis were consecutively enrolled into the study after a written informed consent to participate in the study and for HIV testing. Abdominal tuberculosis was defined as *M. tuberculosis* infections involving the gastrointestinal tract, peritoneum, or intra-abdominal solid organs [8,9]. Patients who failed to give proper history and those without next of kin to consent for the study were excluded from the study. Patients who failed to consent for HIV infection testing were also excluded from the study. They had been admitted either to the surgical, medical, pediatric or emergency department according to the age and clinical presentation. The criteria for diagnosis of abdominal tuberculosis were clinical suspicion, laboratory findings, operative findings, proven histologically, demonstration of AFB and response to anti-tuberculosis drugs. In all cases, assessment was done by detailed history and physical examination and relevant investigations. Relevant preoperative investigations included hemoglobin levels, packed cell volume, serum electrolytes, urea and creatinine, blood grouping and cross-matching and ESR. Patients were also screened for HIV testing using Tanzania HIV Rapid Test Algorithm [17] and CD 4+ count using FACS or FACSCALIBUR from BD Biosciences USA. A determination of CD 4 count was only performed in HIV positive patients. Radiological investigations included Chest X-ray, abdominal X- ray and abdominal ultrasonography. Patients with normal chest X- rays but had symptoms and signs of abdominal tuberculosis were considered to have primary abdominal tuberculosis. Abdominal ultrasound and CT scan were also performed in some patients suspected to have associated abdominal collections or masses.

All patients were treated either surgically or non-surgically by anti tuberculosis therapy. The operations were performed either by a consultant surgeon or a senior resident under the direct supervision of a consultant surgeon. Intraoperative tissue biopsy was taken for histopathological studies; a portion of the tissue was fixed in 10 per cent formalin; routine processing was done as per standard operative procedures and stained with haemotoxylin and eosin. Presence of caseating granulomas surrounded by epitheloid cells, lymphocytes, plasma cells and giant cells were diagnostic of tuberculosis [18,19]. Post-operatively patients were kept nil orally till return of bowl sounds and at that time nasogastric tubes were removed. Final diagnosis and postoperative treatment was dependent on the operative findings and histopathological confirmation. Those found to be tuberculous were started on anti tuberculosis therapy according to the Tanzania National Tuberculosis and Leprosy Programme (NTLP) [20]. The anti tuberculosis therapy given included Isoniazid, Rifampicin, Pyrazinamide, Ethambutol and Streptomycin. All patients had been managed by medical and surgical teams.

Data on each patient were entered into a pro forma prepared for the study. The study variables included socio-demographic (i.e. age and sex, level of education, occupation and area of residence), clinical presentation, HIV status, radiological findings, timing of surgical procedure, operative findings and surgical procedure performed. The variables studied in the postoperative period were postoperative complications, hospital stay and mortality. Patients were followed up for a period of twelve months or till death whichever is earlier.

### Statistical data analysis

The statistical analysis was performed using statistical package for social sciences (SPSS) version 17.0 for Windows (SPSS, Chicago IL, U.S.A).The median + Interquartile Range (IQR) and ranges were calculated for continuous variables whereas proportions and frequency tables were used to summarize categorical variables. Chi-square (*χ*2) test were used to test for the significance of association between the independent (predictor) and dependent (outcome) variables in the categorical variables. The level of significance was considered as *p* < 0.05. Study variable that was found to be statistically significant in univariate analysis were subjected to multivariate logistic regression analysis. Multivariate logistic regression analysis was used to determine predictor variables that predict the postoperative complications, hospital stay and mortality.

### Ethical consideration

Ethical approval to conduct the study was obtained from the CUHAS-Bugando/BMC joint institutional ethic review committee before the commencement of the study. Patients were required to sign a written informed consent for the study and for HIV testing.

## Results

### Socio-demographic profile

**Figure 1:**
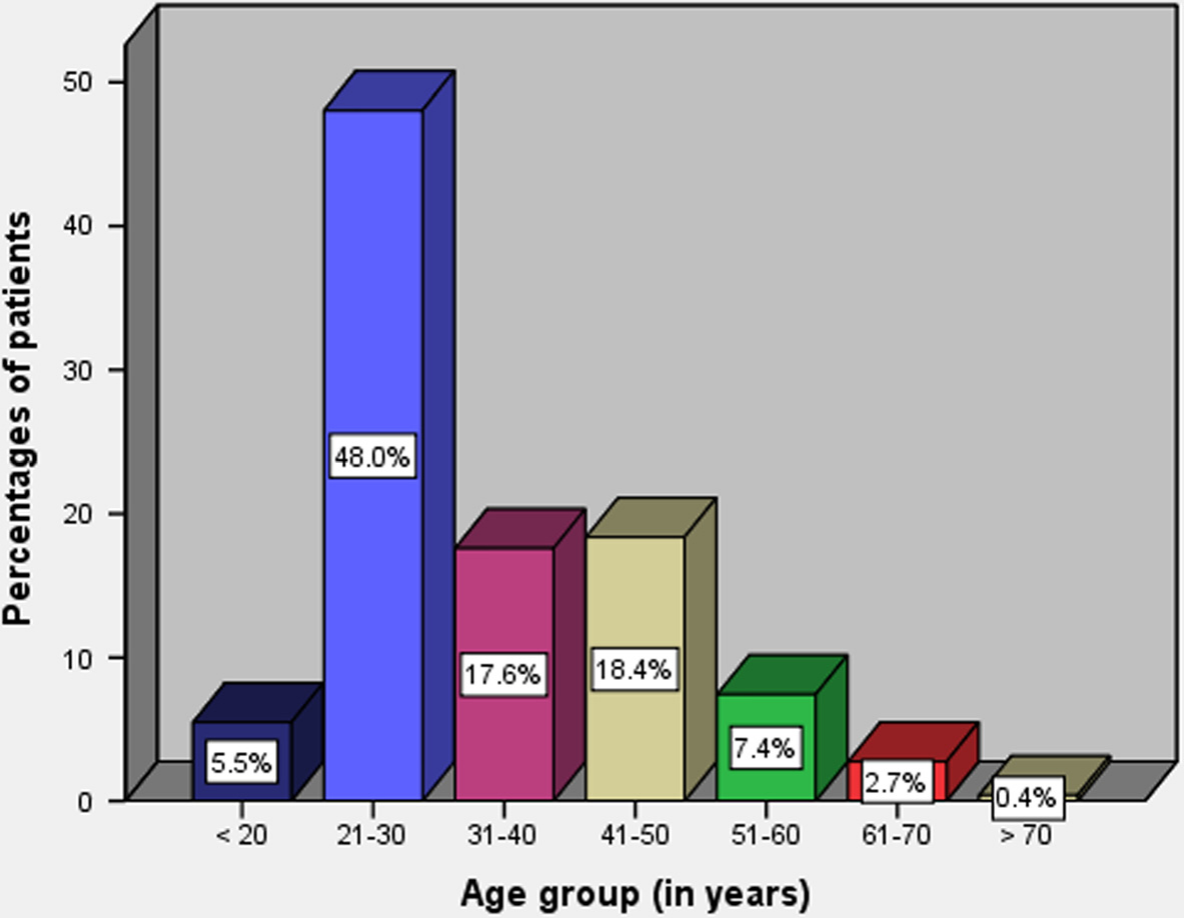
Distribution of patients according to age group.

Out of 256 patients enrolled in the study, 148 (57.8%) were males and 108 (42.2%) were females with a male to female ratio of 1.4: 1. The age of patients ranged from 16 to 68 years with a median age of 28 (IQR = 22 to 38) years. The modal age group was 21–30 years (Figure 1). The majority of patients, 211 (82.4%) had either primary or no formal education and more than eighty percent of them were unemployed. The majority of patients, 198 (77.3%) came from poor families in the rural areas located a considerable distance from the study area. More than ninety percent of our study patients had no identifiable health insurance.

### Clinical presentation of patients with abdominal tuberculosis

Generally, the duration of illness prior to admission in this study varied from 2 days to 3 years with a median of 6 (IQR = 4 to 10) months. The majority of patients, 152 (59.4%) had symptoms of more than 6 months duration at the time of presentation. The commonest presenting symptom was abdominal pain in 240 (93.8%) patients as shown in Table 1. The most common mode of presentation was acute in 182 (71.1%) patients, followed by sub-acute and chronic presentation in 47 (18.4%) and 27 (10.5%) patients respectively. A total of 127 (49.6%) patients presented with intestinal obstruction, 106 (41.4%) with peritonitis, 17 (6.6%) with abdominal masses and 6 (2.3%) patients with multiple fistulae in ano. Co-existing medical illness was recorded in 15 (5.9%) patients. This included diabetes mellitus in five patients, liver cirrhosis in three patients, cancer, hypertension and chronic renal failure in two patients each respectively and toxic goiter in one patient. Forty-eight (18.8%) patients were HIV positive. Of these, 16 (33.3%) patients were known cases on ant-retroviral therapy (ARV) and the remaining 32 (66.7%) patients were newly diagnosed patients. Out of 48 HIV positive patients, 37 (77.1%) had risk factors for HIV infection such as multiple sexual partners (Odd Ratio 4.56, C.I. (2.98- 6.40), p = 0.001) and alcoholism (Odds Ratio 3.51, C.I. (2.17- 12.39), p = 0.011). Past history of treatment for tuberculosis was present in 18 (7.0%) patients and a family history of tuberculosis was found in 9 (3.5%) patients. The majority of patients, 198 (77.3%) had primary abdominal tuberculosis and the remaining 58 (22.7%) patients had associated pulmonary tuberculosis (secondary abdominal tuberculosis).

**Table 1:** Distribution of patients according to clinical presentation

**Clinical presentation**	**Frequency**	**Percentage**
Abdominal pain	240	93.8
Vomiting	204	79.7
Constipation	165	64.5
Weight loss	122	47.7
Abdominal distention	94	36.7
Fever	86	33.6
Diarrhea/constipation	78	30.5
Features of peritonism	70	27.3
Abdominal tenderness/muscle guarding	70	27.3
Abdominal mass	17	6.6
Perianal abscess	6	2.3

### Laboratory, radiological and histological investigations

The median hemoglobin level and ESR were 8.0 (IQR = 4 to 10 g/dl) (range = 4.2-12.6 g/dl) and 56 mm/hour (IQR = 34 to 78mm/hour) (range=32- 145mm/hour) respectively. The hemoglobin level was less than 10 g/dl in 211 (82.4%) patients. Serum creatinine and electrolytes were performed in all patients and revealed low results in 102 (39.8%) and 67 (26.2%) patients respectively. Serological test for HIV infection revealed positive results in forty-eight (18.8%) patients. CD 4+ count was available only in thirty-two patients and their median CD 4+ count was 216 cells/μl (IQR = 154 to 562 cells/μl) (range 54–640 cells/μl). A total of eighteen (56.3%) HIV patients had CD4+ count below 200 cells/μl and remaining 14 (43.7%) patients had CD4+ count of ≥200 cells/μl. No acid-fast bacilli culture of the ascetic fluid was performed. Plain radiography of the abdomen revealed multiple dilated loops of small gut with significant air-fluid levels in erect films in 120 (46.9%) patients. Free air under the right dome of diaphragm was seen in 76 (29.7%) patients. Features suggestive of pulmonary tuberculosis on chest x-rays were found in 58 (22.7%) patients and 12 (4.7%) had radiological features of active pulmonary tuberculosis. Abdominal ultrasound and computed tomography (CT) scan were performed in 142 (55.5%) and 23 (9.0%) patients respectively and revealed abnormal findings suggestive of abdominal TB such as ascites, enlarged lymph nodes, omental thickening, bowel wall thickness and abdominal masses. According to abdominal ultrasound, 34 patients had ascites with fibrinous strands seen in ascitic fluid in 25 (73.5%) patients. Barium studies were performed in 72 (28.1%) patients and common features suggestive of abdominal TB were luminal narrowing with proximal dilatation of bowel loops. Histological examination on biopsy specimen was done in 221(86.3%) patients and revealed presence of non caseating granuloma in 138 (53.9%) patients, central caseation in 56 (21.9%) patients and chronic inflammatory cells infiltration with no definite granuloma in 27 (10.5%) patients. In thirty-five (13.7%) patients with suggestive clinical history and negative diagnostic workup, response to therapeutic trial of anti TB drugs was the basis of diagnosis. In this study, no patient had colonoscopic investigation for intestinal TB. The use of Polymerase Chain Reaction (PCR) to detect *M. tuberculosis* in abdominal tuberculosis was not performed in any of our patients.

### Admission pattern among patients with abdominal tuberculosis

In this study, two hundred and twenty-four (87.5%) patients were admitted to the general surgical wards. The remaining thirty-two (12.5%) patients were treated as out-patients.

### Operative findings, sites of abdominal TB involvement and treatment modalities

**Figure 2:**
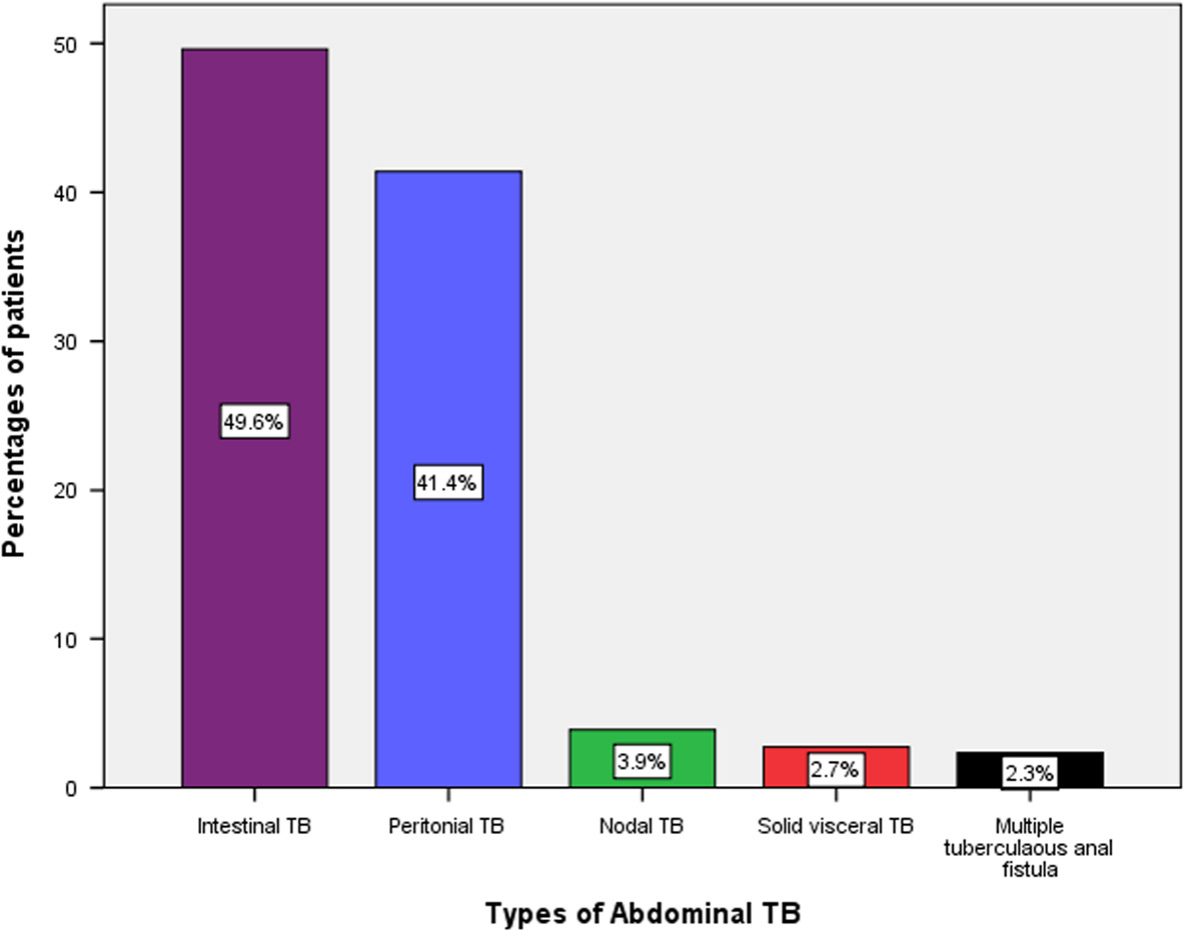
Distribution of patients according to the type of abdominal TB.

A total of 212 (82.8%) patients underwent surgical treatment for abdominal tuberculosis and the remaining 44 (17.2%) patients were treated conservatively with antituberculous therapy. Of those who underwent surgery, 182 (85.8%) were operated on emergency basis while 30 (14.2%) patients had an elective surgery due to failure to resolve with conservative management (i.e. poor response to therapeutic trial of antituberculous drugs). Patients who failed to respond to therapeutic trial of ant-tuberculous drugs underwent surgery and tissue diagnosis was established of tuberculosis in all 30 patients. Operative findings of abdominal tuberculosis are depicted in Table 2. The site of abdominal TB involvement was intestinal in 127 (49.6%), peritoneal in 106 (41.4%), nodal in 10 (3.9%) and solid viscera in 7 (2.7%) patients. The remaining 6 (2.3%) patients had multiple perianal fistulae (Figure 2).

**Table 2:** **Distribution of patients according to operative findings** (**N=212)**

**Operative findings**	**Frequency**	**Percentages**
Bands and adhesions	124	58.5
Strictures	78	36.8
Purulent peritonitis	76	35.8
Bowel perforations	32	15.1
Gross adhesions	26	12.3
Ileo-caecal mass	14	6.6
Enlarged mesenteric lymph nodes with adherent small bowel	10	4.7
Splenic /hepatic mass	7	3.3
Appendicular perforation	6	2.8

Ileo-caecal region was the most common bowel involved in 122 (57.5%) patients, followed by ileum and jejunum in 72 (34.0%) and 12 (5.7%) patients respectively. The colon was involved in 6 (2.8%) patients. Release of bands and adhesions was the most frequent surgical procedure performed in 58.5% of cases (Table 3). Postoperatively all the patients were required to take anti-tuberculous drugs for a period of one year. Patients receiving anti-tuberculous therapy had 4 weekly follow-ups.

**Table 3:** Distribution of patients according to the type of surgical procedure performed (N = 212)

**Type of surgical procedures**	**Frequency**	**Percentage**
Release of bands and adhesions	124	58.5
Segmental bowel resection with end to end anastomosis	56	26.4
Right hemicolectomy with ileo-transverse anastomosis	14	6.6
Repair of bowel perforation	12	5.7
Exploratory laparotomy + biopsy	8	3.8
Appendicectomy	6	2.8
Splenectomy	4	1.9
Ileostomy	1	0.5
Stricturoplasty	1	0.5

### Treatment outcome and follow up of patients

A total of 124 postoperative complications were recorded in 76 (29.7%) patients, of which surgical site infection (SSI) was the most common complication accounting for 37.1% of cases (Table 4). Surgical site infection was statistically significantly associated with HIV positivity (*p*= 0.004) and low CD 4+ count *p* = 0.000).

**Table 4:** Distribution of patients according to postoperative complications (N = 124)

**Postoperative complication**	**Frequency**	**Percentages**
Surgical site infections	46	37.1
Paralytic ileus	12	9.7
Enterocutaneous fistula	10	8.1
Intraabdominal abscess/ peritonitis	8	6.5
Wound dehiscence/ burst abdomen	6	4.8
Keloids	5	4.0

The overall length of hospital stay (LOS) ranged from 1 to 126 days with a median of 32 days (IQR = 16 to 54 days. The LOS for non-survivors ranged from 1–14 days with a median of 6 days (IQR = 3 to 8days. Patients who had post complications stayed longer in the hospital and this was statistically significant (P = 0.022).

A total of 48 (18.8%) patients died. According to multivariate logistic regression analysis, advanced age (≥ 65 years old) (OR = 6.7, 95% CI (2.2- 11.4), *p* = 0.011), co-morbid illness (OR = 11.1, 95% C.I. (7.1- 14.9), p = 0.021), late presentation (OR = 10.3, 95% CI (8.1- 12.8), *P* = 0.029), HIV positivity (OR = 11.4, 95% CI (9.1- 17.9), *p* = 0.001), low CD 4 count (<200 cells/μl) (OR = 3.8, 95%CI (1.3-15.9), p = 0.000) were statistically significantly associated with mortality.

### Follow up of patients

Out of two hundred and eight survivors, 188 (90.4%) patients were discharged well. Twenty (9.6%) patients were discharged against medical advice. No patient in the present study reported to have permanent disabilities. Of the 208 survivors, only 78 (37.5%) patients were available for follow up at twelve months after discharge and the remaining 130 (62.5%) patients were lost to follow up. No patient showed a relapse of disease during this follow-up period. However six patients developed drug induced hepatitis which all of them recovered with modification of drug therapy.

## Discussion

Abdominal tuberculosis constitutes a major public health problem in developing countries and carries significant morbidity and mortality [1,2,12]. In this review, males were slightly more affected than females, an observation which is in accordance with the results of other workers [14,21]. Other authors have reported female predominance [22-25]. Some authors report that the disease is more common in males in the western countries while in developing counties the females predominate [26]. We could not find in literature the reasons for this gender differences.

Abdominal Tuberculosis can affect any age group but is more common in young people at the peak of their productive life [27]. This is reflected in this study as majority of our patients were in the second and third decades of life, which is consistent with other studies [14,28]. The presentation of abdominal tuberculosis in this age group has great economic impact since these are people in their most productive years and this disease imposes a considerable burden on their families and the society as a whole.

In agreement with other studies [12,29-31], the majority of patients inches study came from poor families in the rural areas located a considerable distance from the study area and more than ninety percent of them had no identifiable health insurance. This observation has an implication on accessibility to health care facilities and awareness of the disease.

In keeping with other authors [30-32], the majority of our patients in this study had symptoms of more than 6 months duration at the time of presentation. The reasons for late presentation in this study may be attributed to the fact that the diagnosis of abdominal TB in its initial stages is usually difficult due to vague and non-specific symptoms as a result patients remain undiagnosed and subsequently present late with complications such as intestinal obstruction and bowel perforation with peritonitis. Late presentation in this study may also be attributed to lack of accessibility to health care facilities, lack of awareness of the disease as a result some patients with tuberculous intestinal obstruction may decide to take medications in the pre-hospital period with hope that the symptoms will abate. It is also possible that some clinicians managing the patients initially may not have considered as a possible diagnosis. In resource-poor countries like Tanzania, difficulties in diagnosis of abdominal TB, patient transfer, and inadequate medical treatment often result in delayed presentation to a hospital.

In our study, majority of patients were having acute presentation and were admitted through emergency department with intestinal obstruction and peritonitis requiring emergency exploratory laparotomy. Other authors have also reported similar observations [25,33]. The presence of large number of patients with intestinal obstruction and peritonitis in our series may be attributed to diagnostic delay of abdominal TB leading to development of complications such as intestinal obstruction and peritonitis as a result of bowel perforations. Anal tuberculosis (tuberculous anal fistulae) has been reported to be less uncommon and has a distinct clinical presentation. Tubercular fistulae are usually multiple and recurrent [33-36]. Shukla *et al.*[37] reported that tuberculosis accounted for up to 14 per cent of cases of fistula in ano. In our study, fistula in *ano* was reported in only 2.3% of cases and all of them were multiple.

The majority of patients in this study had primary abdominal tuberculosis and only 22.7% of patients had associated pulmonary tuberculosis (secondary abdominal tuberculosis). The high prevalence of primary intestinal tuberculosis in the present series is in accordance with most of the other studies conducted in developing countries [14,25,38]. Studies from developed countries have shown secondary tuberculosis to be more common [39]. We could not establish the reasons for this geographical variation.

The presence of co-existing medical illness has been reported elsewhere to have an effect on the outcome of patients with tuberculous intestinal obstruction [40]. This is reflected in our study where patients with co-existing medical illness had significantly high mortality rate.

In this study, HIV seroprevalence was found to be 18.8%, a figure that is significantly higher than that in the general population in Tanzania (6.5%) [41]. High HIV seroprevalence in our study may be attributed to high percentage of the risk factors for HIV infection reported in our study population. However, failure to detect HIV infection during window period and exclusion of some patients from the study may have underestimated the prevalence of HIV infection among these patients. HIV infection has been reported to increase the risk of surgical site infection and mortality [42]. In our study, the rate of surgical site infections and mortality was found to be significantly higher in HIV positive patients than in non HIV patients.

Radiological investigation is the mainstay in making presumptive diagnosis of abdominal TB, this include chest x-rays, ultrasound or CT scan of the abdomen and barium studies [43,44]. Of all the radiological investigations, CT scan of the abdomen, which is a costly investigation, gives a better view of intestinal and extra intestinal structures. However, in this review, CT scan of the abdomen was done in only 9.0% of patients, which is in keeping with other studies [45,46]. This was attributed to irregular availability of CT scan due to breakdown or inability of patients to afford. Diagnosis made on the basis of radiology is rapid, easy and less expensive but it is presumptive and cannot exclude completely other diseases like Crohn’s disease and malignancies of solid abdominal viscera [47].

Demonstrating tuberculous granuloma is probably the most important investigation for a definitive diagnosis of abdominal tuberculosis. In our study, histopathology was the basis of diagnosis in 86.3% of patients; however a typical granuloma with caseation was found only in 21.9% of patients in our series. Similar histopathological pattern was reported by Khan *et al.*[48]. The yield of demonstrating tuberculous granuloma has been reported to be high when the specimen is taken surgically or through laparoscopy than when it is taken colonoscopically. On colonoscopic biopsies, if granuloma is non-caseating, interpretation is difficult because Crohn’s disease cannot be excluded [45].

Many authors advocated therapeutic trial with anti tubercular therapy but it should not be encouraged routinely as it may delay the diagnosis of malignancy, lymphoma and Crohn’s disease [45,49]. In the present study, response to therapeutic trial of anti TB drugs was the basis of diagnosis in 13.7% of patients with suggestive clinical history and negative diagnostic workup. This figure is high than 2.0% reported by Khan *et al.*[48]. In the literature up to 40% of patients were given therapeutic trial of anti TB drugs [46].

In keeping with other reports [14,32,48,49], intestinal TB was the most predominant form of abdominal TB in this series and accounted for 49.6% of patients. The majority of patients in this review had ileocaecal region involvement. This is in agreement with other reviews on abdominal tuberculosis in which intestinal type of abdominal tuberculosis ranged from 50%-78% [48,50]. It is postulated that ileocaecal involvement is due to either physiological stasis, large surface area of this part of the intestine, complete digestion of food and abundant lymph nodes in the region [11]. It has been shown that the M cells associated with Peyer’s patches can phagocytes BCG bacilli [51].

Bands and adhesions were the most common operative findings in this study. Similar operative findings were reported by Ali *et al.*[52], but in sharp contrast to other authors who reported bowel strictures as the most frequent intra-operative findings [31,32].

In our series, release of bands and adhesions was the most frequent surgical procedure performed followed by segmental bowel resection with end to end anastomosis. Similar surgical treatment pattern was reported by other writers also [25,52]. This is in contrast to that reported by Akbar *et al.*[32] who reported stricturoplasty as the most common surgical procedure performed. As reported by others [31,32,48,52], anti-tuberculous therapy was prescribed in all the tubercular patients postoperatively.

In agreement with other studies [25,30], surgical site infection was the commonest postoperative complications in the present study attributing this to HIV seropositivity and low CD 4 count.

In this study, the overall median duration of hospital stay was 32 days which is higher than that reported by other authors [30-32]. This can be explained by the presence of large number of patients with postoperative complications in our study. However, due to the poor socio-economic conditions in Tanzania, the duration of inpatient stay for our patients may be longer than expected.

Our mortality rate of 18.8% was significantly higher than that reported by other authors [30,48]. High mortality rate was reported in patients with advanced age, co-morbid illness, HIV positivity with low CD 4 count and those who presented late to the hospital. Addressing these factors responsible for high mortality in our patients is mandatory to be able to reduce mortality associated with this disease.

Discharge against medical advice is a recognized problem in our setting. Similarly, poor follow up visits after discharge from hospitals remain a cause for concern. In this study, the follow up of our patients was generally poor as more than sixty percent were lost to follow up. These issues are often the results of poverty, long distance from the hospitals and ignorance. Delayed presentation, lack of theatre space, delayed histopathological confirmation of abdominal tuberculosis and the large number of loss to follow up was the major limitations in this study.

However, despite these limitations, the study has provided local data that can be utilized by health care providers to plan for preventive strategies as well as establishment of management guidelines for these patients. The challenges identified in the management of patients with abdominal tuberculosis in our environment need to be addressed, in order to deliver optimal care for these patients.

## Conclusion

Abdominal tuberculosis constitutes a major public health problem in our environment and presents a diagnostic challenge requiring a high index of clinical suspicion. Young age at presentation, delayed presentation, poverty and high morbidity and mortality are among the hallmarks of the disease in this region. These challenges need to be addressed in order to deliver optimal care for these patients. Early diagnosis, early antituberculous therapy and surgical treatment of the associated complications are essential for survival.

## Competing interests

The authors declare that they have no competing interests. The study had no external funding. All operational costs were met by authors.

## Authors’ contributions

PLC participated in study design, literature search, data analysis, manuscript writing, editing and submission of the manuscript. MDM, SEM, PR, HJ and JBM participated in data analysis, manuscript writing & editing. All the authors read and approved the final manuscript.

## Pre-publication history

The pre-publication history for this paper can be accessed here:

http://www.biomedcentral.com/1471-2334/13/270/prepub
